# A genome-wide survey with different rapeseed ecotypes uncovers footprints of domestication and breeding

**DOI:** 10.1093/jxb/erx311

**Published:** 2017-09-11

**Authors:** Dayong Wei, Yixin Cui, Yajun He, Qing Xiong, Lunwen Qian, Chaobo Tong, Guangyuan Lu, Yijuan Ding, Jiana Li, Christian Jung, Wei Qian

**Affiliations:** 1 College of Agronomy and Biotechnology, Southwest University, Chongqing 400715, China; 2 College of Horticulture and Landscape Architecture, Southwest University, Chongqing 400715, China; 3 School of Computer and Information Science, Southwest University, Chongqing 400715, China; 4 Department of Plant Breeding, IFZ Research Centre for Biosystems, Land Use and Nutrition, Justus Liebig University, Giessen, Germany; 5 Oil Crops Research Institute, Chinese Academy of Agricultural Sciences, Wuhan 430062, China; 6 Plant Breeding Institute, Christian Albrechts University of Kiel, Olshausenstr. 40, D-24098 Kiel, Germany

**Keywords:** *Brassica napus*, domestication, ecotype, linkage disequilibrium, plant breeding, selective sweep

## Abstract

Rapeseed (*Brassica napus* L.) is an important oilseed crop. Despite a short period of domestication and breeding, rapeseed has formed three diverse ecotype groups, namely spring, winter, and semi-winter. However, the genetic changes among the three ecotype groups have remained largely unknown. To detect selective signals, a set of 327 accessions from a worldwide collection were genotyped using a *Brassica* array, producing 33 186 high-quality single nucleotide polymorphisms (SNPs). Linkage disequilibrium (LD) was unevenly distributed across the genome. A total of 705 (78.2%) weak LD regions were found in the A subgenome, whereas 445 (72.6%) strong LD regions were in the C subgenome. By calculating the nucleotide diversity and population differentiation indices, a total of 198 selective sweeps were identified across ecotype groups, spanning 5.91% (37.9 Mb) of the genome. Within these genome regions, a few known functional genes or loci were found to be in association with environmental adaptability and yield-related traits. In particular, all 12 SNPs detected in significant association with flowering time among accessions were in the selection regions between ecotype groups. These findings provide new insights into the structure of the *B. napus* genome and uncover the footprints of domestication and breeding.

## Teaser

In 198 selective sweeps we found functional genes or loci, such as FT and resistance, associated with environmental adaptability and yield-related traits.

## Introduction

Crop domestication and improvement is a selection process to adapt to different growth conditions and satisfy human preferences. A domestication allele and its neighboring neutral alleles suffer from strong positive selection pressure and produce selective sweeps to fix the domestication allele by reducing or eliminating variance among the nucleotides in neighboring DNA of a domestication locus (Purugganan and Fuller, 2009; Meyer and Purugganan, 2013). Recently, a few domesticated genes with large genetic effects have been identified in various crops, enhancing our understanding of domestication and improvement. For example, *sh4* is responsible for the reduction of grain shattering in rice (Li *et al.*, 2006; Zhang *et al.*, 2009), *Q* controls shattering and free-threshing in wheat (Simons *et al.*, 2006), *DEP1* enhances grain yield in rice (Huang *et al.*, 2009), *KRN4* controls kernel row number in maize (Liu *et al.*, 2015), *fw2.2* influences fruit weight in tomato (Lin *et al.*, 2014), and *CsaBCH1* enhances nutritional value in cucumber (Qi *et al.*, 2013).

The majority of domestication traits are controlled by many genes distributed over the whole genome (Cavanagh *et al.*, 2013; Lin *et al.*, 2014). Next-generation DNA sequencing technologies and SNP (single nucleotide polymorphism) arrays offer new and powerful tools to detect genomic footprints for those complex domestication traits. By genomic resequencing of diverse varieties, Mace *et al.* (2013) found that the genes in selection regions were enriched in functional categories in response to auxin and biotic and abiotic stress in sorghum. Xie *et al.* (2015) found that the genes in the regions under selection pressure are enriched in the regulatory pathways associated with flowering time (FT; heading time), nitrogen assimilation, and hormones in rice. More recently, Cheng *et al.* (2016) found that the genes related to four phytohormones (cytokinin, auxin, gibberellins, and jasmonic acid) were significantly enriched in selective sweeps of the leaf-heading morphotypes of both *Brassica rapa* and *B. oleracea*. Wang *et al.* (2014) identified genetic changes during modern breeding of rapeseed using a *Brassica* SNP array. Another study conducted by Wang *et al.* (2017) showed asymmetric subgenome selection and *cis*-regulatory divergence during cotton domestication.

Rapeseed (*Brassica napus* L., AACC) was derived from an interspecific hybridization between *B. rapa* (AA) and *B. oleracea* (CC), ~7500 years ago (Chalhoub *et al.*, 2014). As compared with its parental species, the domestication history of rapeseed is relatively short. Rapeseed was documented as a winter crop in Europe 400 years ago, which has a biennial life cycle and strong vernalization requirement, and as a spring crop without vernalization ~300 years ago (Gómez-Campo and Prakash, 1999). After introduction and improvement to adapt to the local environment, rapeseed was widely grown in China as a semi-winter crop, which has a biennial life cycle and a moderate vernalization requirement (Liu, 2000). In the 1960s and 1970s, the crop was introduced into Canada and Australia as a spring crop (Chen *et al.*, 2008). Presently, rapeseed is one of the most important oilseed crops in the world. Despite a short history of domestication and breeding, substantial genetic diversity was found among the three ecotype groups (Diers and Osborn, 1994; Becker *et al.*, 1995; Wang *et al.*, 2014). However, those loci and their genomic regions under selection remained poorly understood. The aim of this study is to understand genomic footprints of domestication and breeding among the three ecotype groups. We genotyped 327 accessions from a worldwide collection with a whole-genome SNP array, and found that the linkage disequilibrium (LD) decay was not evenly distributed throughout the genome of rapeseed. A few genes associated with yield-related and environmental adaptability traits, including FT and resistance were harbored in selective sweeps identified across different ecotype groups. Our investigation offers new opportunities to improve rapeseed by targeting those selected loci.

## Materials and methods

### Plant materials and phenotypic evaluation

The experimental set of 327 *B. napus* accessions was comprised of 71 winter ecotype lines from Europe, 60 spring ecotype lines from Europe (37), Canada (7), North Korea (3), Australia (4), and unknown regions (9) randomly selected from the ERANET-ASSYST *B. napus* diversity set (Bus *et al.*, 2011), and 196 Chinese semi-winter accessions (Qian *et al.*, 2014) (Supplementary Table S1 at *JXB* online). The accessions were sown in the middle of September in the experimental field of the Southwest University, Chongqing, China (29°33'N, 106°34'E), in four consecutive years from 2012 to 2015. A randomized complete block design was performed with two replications. Each plot consisted of 24 plants, with 30 cm between rows and 25 cm within rows. Field management followed essentially the normal agricultural practice. FT was recorded as days from sowing to flowering when 50% of the plants in a plot had reached the flowering stage.

### SNP genotyping

Genomic DNA was extracted from the bulked young leaves of 24 plants of each genotype using the TIANGEN^®^ plant genomic extraction kit (DP305-03) (Beijing, China). Accessions were genotyped using the *Brassica* 60K Illumina Infinium^®^ SNP array (Edwards *et al.*, 2013), according to the Infinium^®^ HD Assay Ultra Protocol Guide. Using a local BLASTn search, the SNPs of the array were aligned to the *B. napus* reference genome assembly v4.1 (Chalhoub *et al.*, 2014). Only the top BLAST hits against the reference genome were considered, allowing no less than 50 bp overlap, 90% sequence identity, and no gaps (Clarke *et al.*, 2016).

### Analysis of phylogenetic relationships

The population structure of rapeseed was analyzed with 5700 SNPs [minor allele frequency (MAF) >0.05] evenly distributed across 19 chromosomes by using STRUCTURE v2.3.4 (Pritchard *et al.*, 2000). Five independent simulations having 1 × 10^5^ MCMC (Markov chain Monte Carlo) replications and 1 × 10^5^ burn-ins were performed, with the number of subpopulations (*k*) ranging from 1 to 10. The optimal *k*-value was determined by the log probability of data [LnP(D)] and an ad hoc statistic Δ*k* based on the rate of change of LnP(D) between successive *k* as described by Evanno *et al.* (2005). Principal component analysis (PCA) was performed by TASSEL v5.2 software (Bradbury *et al.*, 2007). The Neighbor–Joining tree was generated by TASSEL’s Cladogram function and visualized using Figtree v1.4.0 (http://tree.bio.ed.ac.uk/software/figtree/).

### Linkage disequilibrium analysis

LD was estimated by measuring the *r*^2^ value via the software package TASSEL v5.2. Considering that crossovers and recombination events mainly occur within the 10 kb region (Paape *et al.*, 2012), the highest or lowest 5% of *r*^2^ within the 10 kb region (*P*<0.01) was defined as strong or weak LD, respectively. The homoelogous regions were detected for the LD region between the A and C subgenomes using the LAST web service (Kielbasa *et al.*, 2011) with the default parameter, match/mismatch=+1/–1, gap exist/extend= –7/–1 (Frith and Noé, 2014).

### Identification of selective sweep

During crop domestication and breeding, both natural and artificial selection result in the reduction or elimination of variance in the selective sweeps. In order to detect those domestication footprints, using a sliding window approach (100 kb windows sliding in 10 kb steps), we calculated the nucleotide diversity (π) indicating the genomic signals of diversity within ecotype groups and the π ratio between ecotype groups with powermarker v3.25 (Liu and Muse, 2005), the selection statistics index (Tajima’s *D*) showing the allele frequency distribution relative to neutral expectations in ecotype groups by using TASSEL v5.2 software, and the population differentiation index (*F*_ST_) quantifying levels of differentiation between ecotype groups using Genepop v4.2 software with the default parameter (Rousset, 2008). Since each statistical approach has its own strengths and limitations, combining multiple tests may increase the power and resolution to identify selection signals. According to an empirical procedure described by Li *et al.* (2013), the intersection regions with the top low or high π ratios (corresponding to the 5% left and right tails of the empirical π ratio distribution) and the top high *F*_ST_ values between ecotype groups were identified as selective sweeps in the genome. The genes harbored in these selective sweeps should have been under selection during rapeseed domestication and breeding.

To test whether the population structure influenced the identification of selective sweeps, a permutation test was performed using a Java script written in house by randomly shifting individuals across groups, and calculating *F*_ST_ between groups. We replicated this process 2000 times to assess the significance of an *F*_ST_ value.

To test whether some homoeologous genes with a similar function were possibly involved in ecotype differentiation in various plant species, the genes in the top 5% *F*_ST_ regions were isolated in our SNP data, while differentially expressed genes (DEGs) between winter and spring ecotypes were identified with the rapeseed transcriptome sequencing data set (Lu *et al.*, 2014) through DEGseq of the R package (Wang *et al.*, 2010), and with the Arabidopsis expression microarray data set (Des Marais *et al.*, 2012) using a custom R script with Wilcox rank-sum test (*P*<0.01). Further, we searched for the overlapping homoeologous genes among the three data sets by aligning *B. napus* genes to the Arabidopsis genome with use of BLAST.

### Genome-wide association analysis for flowering time

FT is a very important trait in association with crop domestication and adaptation breeding. In order to verify that loci associated with FT are located in the regions with strong selective signal, we investigated FT in accessions across 4 years. The best linear unbiased prediction (BLUP) for FT was estimated by using an R script based on a linear model as described by Merk *et al.* (2012). Association analysis for FT was carried out using a mixed model (MLM) with PC adjustment, which reduces the biased effects from population structure (Aulchenko *et al.*, 2007). For the MLM analysis, we used the equation: y=Xα+Pβ+Kµ+e, where y represents phenotype, X represents genotype, P is the PCA matrix instead of the Q matrix, and K is the relative kinship matrix. Xα and Pβ represent fixed effects, and Kμ and e represent random effects (Yu *et al.*, 2006; Zhang *et al.*, 2010). The threshold of the genome-wide association study (GWAS) was set to *P*-value <4.86 × 10^–5^ (1/total SNPs used). The region of interest was defined using the LD decay around the most significantly associated SNP markers, which extended until *r*^2^ decayed to <0.5 on both sides or 200 kb on each side of the SNP peak (Zhang *et al.*, 2015).

### Enrichment analysis

The function of selected genes was analyzed by using the Kyoto Encyclopedia of Genes and Genomes (KEGG) database (http://www.kegg.jp/blastkoala/), and pathway analysis was determined by KOBAS version 2.0 (http://kobas.cbi.pku.edu.cn/). Gene Ontology (GO) for selected genes was studied with the web toolkit agriGO (http://bioinfo.cau.edu.cn/agriGO/) (Du *et al.*, 2010) with the Fisher’s exact test method (*P*<0.05) and Hochberg multitest adjustment method [false discovery rate (FDR) <0.05].

## Results

### Genome structure of different rapeseed ecotypes

**Fig. 1.**
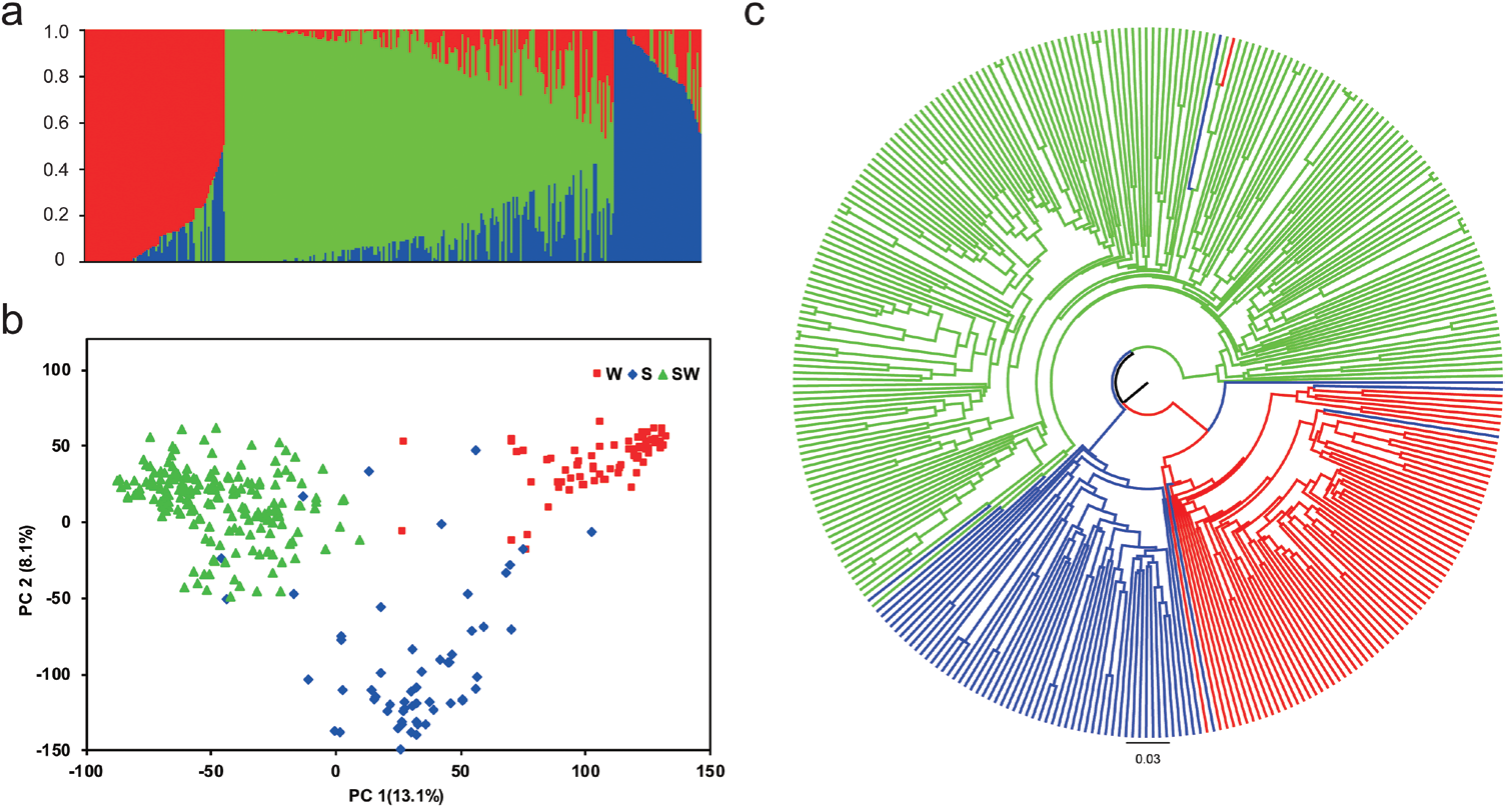
Phylogenetic relationships among 327 *Brassica napus* accessions revealed with a *Brassica* 60K SNP array. (a) Bayesian clustering. (b) Principal component analysis. (c) A Neighbor–Joining phylogenetic tree. Winter (W), semi-winter (SW), and spring (S) ecotypes are drawn in red, green, and blue colors, respectively.

Of the 52 157 SNPs from the *Brassica* SNP array, 36 241 SNPs turned out to be informative among 327 accessions (Supplementary Tables S1, S2). Of these, 33 186 high-quality SNPs with an MAF >0.05 were used for genetic structure analysis. The SNP densities across the genome averaged 8 and 4 SNPs per 100 kb in the A and C subgenomes, respectively, ranging from 2 SNPs on C09 to 10 SNPs on A10 per 100 kb (Supplementary Table S3). Using a set of 5 700 SNPs (MAF >0.05) evenly distributed across 19 chromosomes, the accessions were classified into three major genetic groups by the Bayesian clustering program with the highest score of Δ*k* at *k*=3 (Fig. 1a), almost in accordance with the classification of growth habit of the accessions. The result of classification was verified by PCA and a Neighbor–Joining tree based on all the SNPs in the collection (Fig. 1b, c). Moreover, we found clear evidence for genetic introgression from other gene pools. Some of the semi-winter accessions shared alleles with winter and spring accessions, while only a few of the winter and spring accessions possessed genome introgressions from the semi-winter accessions (Fig. 1a).

**Fig. 2.**
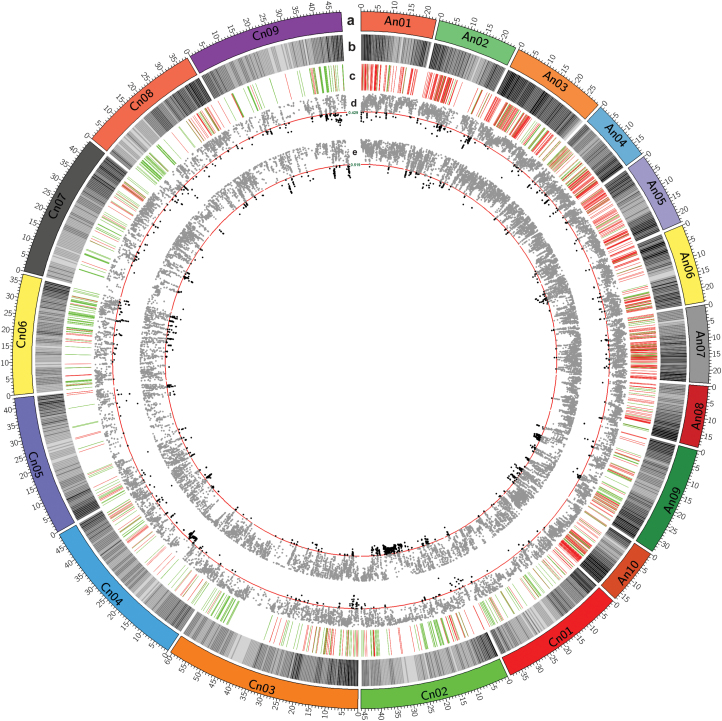
Concentric circles of population differentiation among ecotype groups of *Brassica napus*. (a) Chromosome; (b) heat map view of genes; (c) distribution of weak LD regions (red) and strong LD regions (green) in 10 kb window regions across the whole genome; (d) scatter spots distribution of *F*_ST_ in semi-winter versus winter/spring ecotype groups; (e) scatter spots distribution of *F*_ST_ in winter versus spring ecotype groups. The top 5% of *F*_ST_ values above the threshold line (red) were emphasized with a dark spot.

We also analyzed the LD throughout the whole genome. The average distance over which LD decayed to half of its maximum value was 150–200 kb. However, the size of LD decay varied greatly across chromosomes (Supplementary Fig. S1). The average distance of LD decay in the C subgenome was 10-fold longer (3.5–4 Mb) than that in the A subgenome (Supplementary Fig. S2). Within each 10 kb window of adjacent SNPs, we detected 902 weak LD regions at the bottom 5% of *r*^2^ (covering 0.61% of the genome) and 613 strong LD regions at the top 5% of *r*^2^ (covering 0.32% of the genome) (Supplementary Table S4). Among these, 705 (78.16%) weak LD regions are located in the A subgenome, whereas 445 (72.59%) strong LD regions are located in the C subgenome (Fig. 2). We also found that 101 (51.3%) weak LD regions in the C subgenome were homoeologous to 267 (37.9%) weak LD regions in the A subgenome, and that 205 (46.1%) strong LD regions in the C subgenome were homoeologous to 78 (46.4%) strong LD regions in the A subgenome (Supplementary Table S5). We found that weak LD regions had higher gene density and GC content whereas the regions with strong LD were enriched with retroelements and DNA transposons (Supplementary Table S6).

### Selection signals and population differentiation between semi-winter and winter/spring ecotypes in rapeseed

**Fig. 3.**
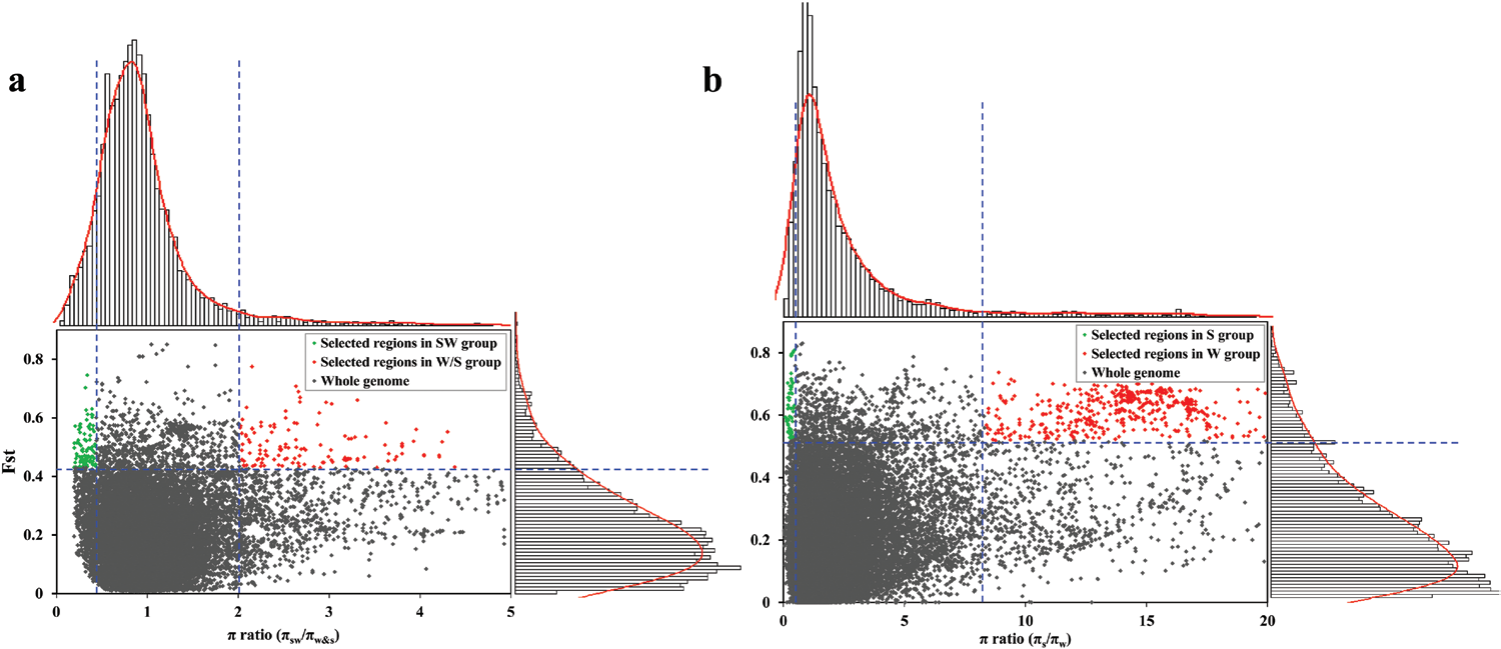
Selective signals in the whole genome between different rapeseed ecotypes. (a) Comparison between semi-winter (SW) and winter/spring (W/S) ecotypes. (b) Comparison between spring (S) and winter (W) ecotypes. The π ratios (π_sw_/π_w&s_ and π_s_/π_w_) and *F*_ST_ values were calculated in a sliding window approach with 10 kb steps in a 100 kb window. The dashed blue lines correspond to the 5% left and right tails of the empirical π ratio distribution, where the π ratios are 0.43 and 2.02 for semi-winter and winter/spring groups (a) and 0.48 and 8.35 for spring and winter groups (b), respectively. Data points located to the left and right of the vertical dashed blue lines and above the horizontal dashed blue line [the 5% right tail of the empirical *F*_ST_ distribution, where *F*_ST_ is 0.429 in (a) and 0.519 in (b)] are indicative of selected regions for the SW group (green dots) and W/S group (red dots) in (a), and for the S group (green dots) and W group (red dots) in (b).

Semi-winter ecotype rapeseed was derived from winter and spring rapeseed no more than 100 years ago, but its genetic structure was diverged from the founder ecotypes as a consequence of breeder selection (Liu, 2000). To elucidate the genomic footprints left by selection, we searched for selection signals between semi-winter and winter/spring ecotypes. Setting a threshold of the 5% top *F*_ST_, we identified 313 genome regions with strong population differentiation between semi-winter and winter/spring ecotypes. Our result was further supported by a permutation test (FDR <0.01) (Fig. 2). Among those genome regions, 66 and 51 regions were indicative of selective sweeps in winter/spring and semi-winter rapeseed, respectively (Fig. 3a). All together, these regions cover 1.52% (9.76 Mb) and 1.41% (9.04 Mb) of the assembled genome, and harbor 1202 and 1045 genes in winter/spring and semi-winter rapeseed, respectively. Interestingly, selective sweeps of winter/spring rapeseed are evenly distributed across the A (45%) and C (55%) subgenomes, while the majority of selective sweeps in semi-winter rapeseed lie in the C subgenome (82%) (Supplementary Tables S7, S8).

We further examined if the genes under selective pressure belong to specific functional categories. The annotated genes within the selective sweeps were subjected to GO analysis. We reasoned that genes responding to environmental cues are well represented within these regions of the genome, because changes of allele frequencies drive adaptive evolution and shape phenotypic variation between ecotypes with a divergent life cycle under different environmental conditions (semi-winter versus spring/winter ecotypes). GO analysis indicates an over-representation of genes involved in environmental adaptation (e.g. response to cold, vernalization response, and response to temperature) and development (e.g. cellular component assembly, morphogenesis, cell growth, and multicellular organismal development) (FDR <0.05) (Supplementary Table S9).

**Fig. 4.**
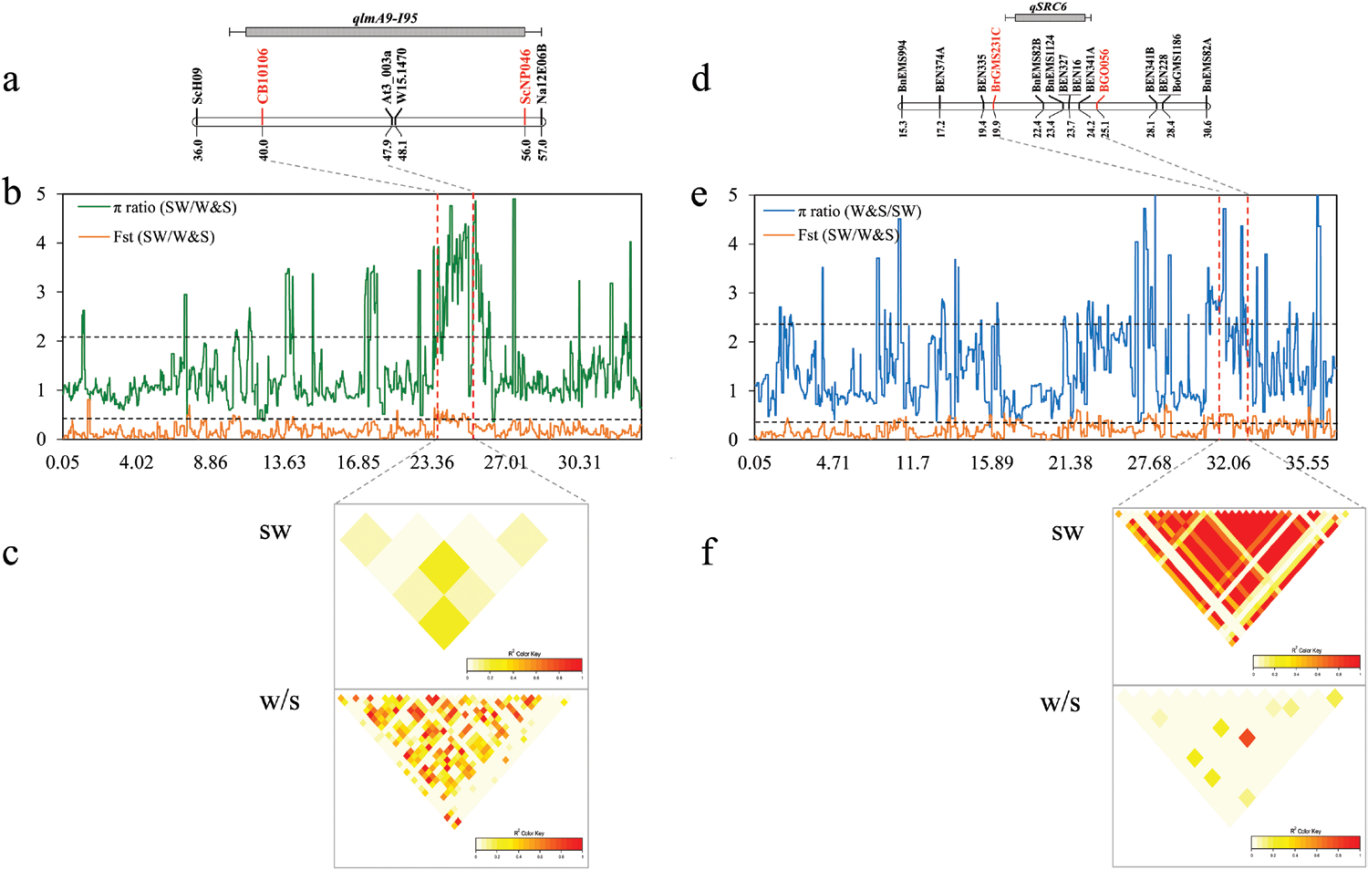
Selective sweep for the blackleg resistance quantitative trait loci (QTLs) on chromosome A09 and the *Sclerotinia* resistance QTLs on chromosome C06. (a and d) Map positions of the blackleg resistance QTL *qLmA9-I95* (Delourme *et al.*, 2008) and the *Sclerotinia* resistance QTL *qSRC6* (Wu *et al.*, 2013). (b and e) Empirical π ratio and *F*_ST_ distributions within the QTL region; the black dotted line indicates the threshold value of the top 5% π ratio and *F*_ST_. (c and f) Linkage disequilibrium pattern of 196 semi-winter (SW) and 131 winter/spring (W/S) ecotypes in the region of the selective sweep; the color key indicates the *r*^2^ values.

Here we present two examples to show diverse selection between ecotype groups. Blackleg (*Leptosphaeria maculans*) is the most devastating disease in the regions where winter and spring rapeseed are found, whereas white mold (*Sclerotinia sclerotiorum*) is a predominant disease in semi-winter rapeseed (Delourme *et al.*, 2008; Sharma *et al.*, 2015). We found that three linked selective sweeps on chromosome A09 (W&S-29, W&S-30, and W&S-31; from 23.6 Mb to 25.4 Mb) are located with a known blackleg resistance quantitative trait locus (QTL) region (*qLmA9-I95*) explaining 4.8–15.2% of the phenotypic variation (Delourme *et al.*, 2008) (Fig. 4a, b). The region displayed strong and extensive LD, a significant negative Tajima’s *D* value (*D*= –0.427), and low genetic diversity (π=0.098) in winter and spring rapeseed group, but not in the semi-winter ecotype group (Fig. 4a–c; Supplementary Table S7). Likewise, a major locus (*SRC6*) explaining 29.01–32.61% of the phenotypic variation for *Sclerotinia* resistance across multiple environments (Wu *et al.*, 2013; Wei *et al.*, 2016) was located within a 1.5 Mb (from 30.85 Mb to 32.28 Mb) selective sweep region (SW-27) on chromosome C06 of the semi-winter group (Fig. 4d, e). This region is flanked by the markers Bn-scaff_23957_1-p270744 and Bn-scaff_16397_1-p622895, where a high genetic differentiation value (*F*_ST_=0.534) and π ratio (π_w&s_/π_sw_=4.72) were observed between semi-winter and winter/spring groups (Figs.4d–f; Supplementary Table S7). To conclude, here we present two genome regions where divergent selection for pathogen resistance has left footprints of breeding for adaptation to certain geographical environments.

### Selection signals and population differentiation between winter and spring ecotypes in rapeseed

Historically, spring and winter rapeseed diverged ~300 years ago (Gómez-Campo and Prakash, 1999; Prakash *et al.*, 2011). We detected 27 and 54 selective regions in spring and winter ecotypes, respectively (Fig. 3b; Supplementary Table S10). These regions cover 4.39 Mb and 14.69 Mb, and harbor 482 and 1 184 genes in spring and winter ecotypes, respectively (Supplementary Table S11). Genes controlling environmental adaptation should have undergone strong selection during domestication and breeding. As expected, GO enrichment analysis revealed strong bias towards genes involved in environmental adaptation and yield-related traits (e.g. response to abiotic and biotic stress or endogenous stimuli), other external factors (e.g. light, jasmonic acid, and nitrogen metabolism), development, carbohydrate biosynthesis, and post-embryonic development (FDR <0.05) (Supplementary Table S12).

We compared our data with the map positions of 61 QTLs for yield-related traits which were earlier identified in a population derived from a winter by spring rapeseed cross (Quijada *et al.*, 2006; Udall *et al.*, 2006). We found that 21 QTLs (34.4%) for seed yield, test weight, seed weight, plant height, days to flowering, lodging, and oil content are located within 33 selective sweep regions (Supplementary Table S13), which demonstrates how breeders and farmers have altered allele frequencies in the past centuries.

**Fig. 5.**
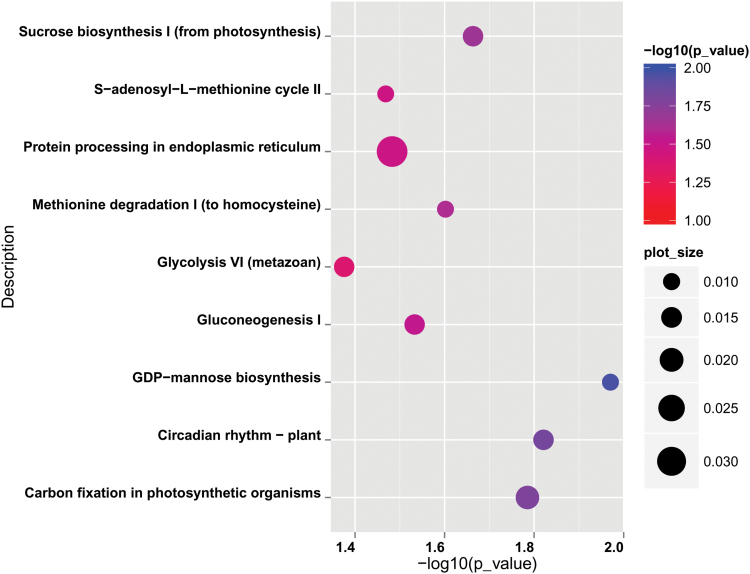
KEGG enrichment of 202 overlapping differential genes between spring and winter ecotypes in rapeseed and Arabidopsis. The enriched pathways are listed on the left. Colors denotes the –log_10_^(*P*-value)^. The size of each bubble reflects the ratio of genes enriched in each pathway to 202 genes.

The annual and biennial life cycles widely exist in various plant species. We hypothesized that some homologous genes accounting for population differentiation should exhibit a similar differential expression pattern between spring and winter ecotypes in various plants. To test this hypothesis, we performed an *in silico* study between rapeseed and Arabidopsis. We aligned 4 304 genes (3 457 analogous Arabidopsis genes) from 228 genome regions differing between winter and spring rapeseed. These genes were then compared with 4 247 DEGs (Arabidopsis homologous genes) of rapeseed detected by transcriptome sequencing (Lu *et al.*, 2014), and 5 500 DEGs from Arabidopsis detected by expression microarray analysis (Des Marais *et al.*, 2012) (Supplementary Tables S14–S16). We detected a subset of 202 overlapping genes in the three data sets (Supplementary Fig. S3; Supplementary Table S17), which were allocated to nine functional categories (*P*-value <0.05) (Fig. 5). Most of the functional categories were found to be involved in energy metabolism, such as sucrose biosynthesis, protein processing in the endoplasmic reticulum, gluconeogenesis 1, GDP-mannose biosynthesis, and carbon fixation in photosynthetic organisms. Interestingly, the pathway of circadian clock regulation was enriched, which was reported in association with vigor and fitness (Dodd *et al.*, 2005; Xie *et al.*, 2015).

### Genome-wide association studies for flowering time

**Fig. 6.**
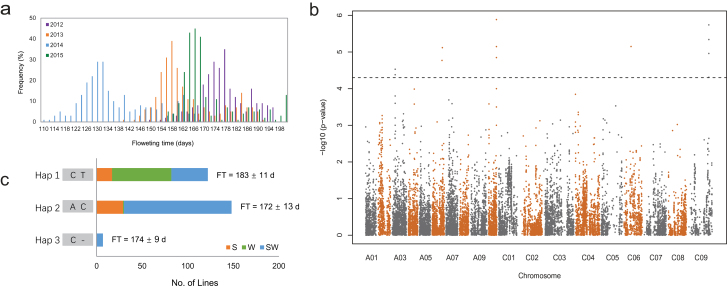
Genome-wide association study (GWAS) and haplotype analysis for flowering time (FT). (a) The frequency distribution of FT across 4 years. (b) Manhattan plots of GWAS for FT. The black dotted line represents the significant level, −log10(*P*-value) >4.3. (c) Haplotype analysis for FT in two SNPs (6 450 533 and 6 450 763) on chromosome A03, linked with *BnaA03g13630D* (homologous to Arabidopsis *Ath.FLC*). S, W, and SW indicate the spring, winter, and semi-winter rapeseed group, respectively.

Flowering time is an important regulatory factor of plant adaptability. We performed GWASs for FT among 327 accessions of rapeseed across four consecutive years. A wide variation in FT was detected among accessions, ranging from 107 d to 204 d (Fig. 6a). As expected, the latest flowering genotypes belong to the winter rapeseed group.

In total, 12 SNPs forming five haplotype blocks on chromosomes A03, A06, A10, C06, and C09 were significantly associated with FT, explaining 4.8–11.4% of the phenotypic variance per SNP (Fig. 6b; Supplementary Table S18). We found strong selection signals in the regions harboring these SNPs loci, which exhibited a high π ratio and/or *F*_ST_ between the three ecotype groups (Supplementary Table S18). Among these, three haplotype blocks were closely linked with the known homologous genes of FT, *BnaA03g13630D*, *BnaA06g28900D*, and *BnaA10g24140D* which are homologous to *Ath.FLC* (*AT5G10140*), *Ath.FRI* (*AT5G27230*), and *Ath.ADC2* (*AT4G34710*), respectively (Supplementary Table S18) (Alcázar *et al.*, 2005; Mendez-Vigo *et al.*, 2011). Significant differences in FT were detected among haplotypes in the five haplotype blocks (Fig. 6b; Supplementary Table S18). For example, two SNPs in A03 (6 450 533 and 6 450 763), linked with *BnaA03g13630D* (homologous to Arabidopsis *Ath.FLC*), formed three haplotypes with significantly different FT (Hap 1–Hap 3) (Fig. 6c; Supplementary Table S18). Most of winter lines (65 out of 71) carried the Hap 1 haplotype (average FT 183 d), while 118 semi-winter accession ecotypes (72%) were of type Hap 2 (average FT 172 d) (Fig. 6c).

## Discussion

Crop breeding has significantly improved crop adaptation to different growth conditions and human preferences. Although rapeseed was introduced from Europe to other parts of the world ~100 years ago, its genome has been diversified into three ecotype groups with diverse genetic basis (Gómez-Campo and Prakash, 1999). The recent availability of reference genome sequences from *Brassica* crops (Wang *et al.*, 2011; Chalhoub *et al.*, 2014; Liu *et al.*, 2014; Yang *et al.*, 2016) allows detailed dissection of the genomic alterations during domestication and breeding. In the present study, we identified 198 selective sweeps by analyzing the whole genome with a *Brassica* DNA microarray, where a number of known genes or loci associated with environmental adaptability and yield-related traits are harbored.

The knowledge about selective sweeps provides insights and targets for utilization of exotic germplasm. Rapeseed breeding suffers from low genetic diversity (Seyis *et al.*, 2003). Exotic germplasm can serve as a valuable source to broaden the genetic basis since it harbors favorable alleles, which are not present in the current rapeseed pool (Quijada *et al.*, 2006; Udall *et al.*, 2006). QTLs for environmental adaptability and yield-related traits were identified in biparental mapping populations from diverse ecotypes in rapeseed (Quijada *et al.*, 2006; Udall *et al.*, 2006; Chen *et al.*, 2007; Long *et al.*, 2007; Radoev *et al.*, 2008; Basunanda *et al.*, 2010; Shi *et al.*, 2011; Raman *et al.*, 2014; Wei *et al.*, 2014). Moreover, new genetic variation is needed to increase the heterotic potential of rapeseed hybrids. Several studies have demonstrated that hybrids between different rapeseed ecotypes exhibit high heterosis (Lefortbuson *et al.*, 1987; Butruille *et al.*, 1999; Qian *et al.*, 2006, 2009). In our study, we detected numerous diverse selective sweeps among ecotype groups in association with environmental adaptability and yield-related traits by analyzing genome structure diversifications between the three ecotype groups. The information of selective sweeps and markers linked with domestication genes will be helpful for molecular breeding utilizing exotic germplasm in rapeseed.

Traditional QTL mapping using biparental populations only detects those QTLs which are polymorphic between the two parents. Here, our study verified that the comparison of genomic structure among diverse subpopulations is an efficient strategy for exploring adaptable genes or loci on a large scale. Similar studies reported in maize and rice also suggested that selective regions are associated with agronomic performance (van Heerwaarden *et al.*, 2012; Xie *et al.*, 2015).

An allopolyploid species contains two or more sets of chromosomes (subgenomes) from related ancestral species. We may ask the question of whether these subgenomes of an allotetraploid could ‘communicate’ with each other during domestication and breeding. Rapeseed (AACC) derived from an interspecific hybrid between *B. rapa* (AA) and *B. oleracea* (CC) is a good model to investigate the evolution of allotetraploid species. High homoeology between A and C genomes (Delourme *et al.*, 2013; Chalhoub *et al.*, 2014) ensures possible communication between A and C subgenomes of rapeseed. By analyzing genome structure among three diverse ecotype groups formed during domestication and breeding, we found an uneven distribution of LD within the two subgenomes. The majority of regions with weak and strong LD were located in the A and C subgenomes, respectively. This indicates higher diversity within the A subgenome than the C subgenome. A possible reason lies in the higher ability of *B. napus* to form a cross with *B. rapa* than with *B. oleracea* (Qian *et al.*, 2006). As a consequence, the gene introgression rate from *B. rapa* into *B. napus* is higher. The introgression of genes from Asian *B. rapa* has further elevated the genetic divergence between semi-winter rapeseed and winter/spring rapeseed (Qian *et al.*, 2006; Mei *et al.*, 2011). Interestingly, we found a high degree of homoeology within weak or strong LD regions between A and C subgenomes. This indicates a high frequency of ‘communication’ between two subgenomes of rapeseed during domestication and breeding. This is in accordance with previous studies where QTLs were localized within regions of homoeologous exchanges (HEs) between A and C subgenomes, as in the case of *Sclerotinia* resistance, FT, seed quality, seed weight, and silique length (Zhao *et al.*, 2006; Harper *et al.*, 2012; Qian *et al.*, 2014; Wei *et al.*, 2014; Fu *et al.*, 2015). Therefore, we propose a possible mechanism of rapeseed evolution, the introgression of genome regions from *B. rapa*, followed by HEs between subgenomes, and artificial selection as well as natural selection leading to fixation of elite loci within the two subgenomes.

### Conclusions

We detected 198 selective sweep regions across ecotype groups. Of these, a number of known functional genes or loci were associated with environmental adaptability and yield-related traits, suggesting that these domestication loci in selective sweeps might have undergone independent and additional selection within ecotype groups for adaptation to the local environment and improvement of yield-related traits. Our findings provide new insights into the structure of the rapeseed genome and uncover the footprints of domestication and breeding.

## Supplementary data

Supplementary data are available at *JXB* online.

Fig. S1. Genome-wide patterns of linkage disequilibrium (LD) across chromosomes in the A (top) and C subgenome (bottom) of *Brassica napus* measured with 33 186 SNPs.

Fig. S2. The average distance of LD decay in the A and C subgenomes of *B. napus*.

Fig. S3. Venny plot of overlapping genes among three data sets in rapeseed and Arabidopsis.

Table S1. List of rapeseed accessions used in this study.

Table S2. Genotype data for 327 accessions in this study (http://pan.baidu.com/s/1kVFPflP).

Table S3. The distribution of SNPs across the 19 chromosomes of the rapeseed genome.

Table S4. Physical position of the weak and strong LDs.

Table S5. Homeologous exchanges between the A and C subgenomes in regions for weak and strong LD.

Table S6. Sequence features of genome regions with weak and strong LD.

Table S7. Regions of selective sweep between the winter/spring and semi-winter groups in rapeseed.

Table S8. Genes within selective sweep regions between semi-winter and winter/spring groups in rapeseed.

Table S9. Functional categories of the genes in the regions of selective sweep between winter/spring and semi-winter groups in rapeseed.

Table S10. Regions of selective sweep between spring and winter groups in rapeseed.

Table S11. Genes within selective sweep regions between spring and winter groups in rapeseed.

Table S12. Functional categories of the genes in the region of selective sweep between spring and winter groups in rapeseed.

Table S13. QTLs were aligned to selective sweeps within the segregation populations derived from a hybrid between spring and winter rapeseed reported by Udall *et al.* (2006) and Quijada *et al.* (2006).

Table S14. A list of 4 304 genes from genome regions differentiating between winter and spring groups in rapeseed.

Table S15. A list of 4 247 differentially expressed genes between winter and spring groups in rapeseed as detected by Lu *et al.* (2014).

Table S16. A list of 5 500 differentially expressed genes between summer and winter ecotypes in *Arabidopsis thaliana* as detected by Des Marais *et al.* (2012).

Table S17. A list of 202 genes overlapping between the three data sets from rapeseed and Arabidopsis.

Table S18. The significant SNPs associated with flowering time across 4 years.

## Supplementary Material

Supplementary Figures

Supplementary Tables

## Abbreviations:

DEG: differentially expressed gene
FDR: false discovery rate
*F*_ST_: population differentiation index
FT: flowering time
GO: Gene Ontology
GWAS: genome-wide association study
HE: homoeologous exchange
KEGG: Kyoto Encyclopedia of Genes and Genomes
LD: linkage disequilibrium
MAF: minor allele frequency
π: nucleotide diversity
PCA: principal component analysis
QTL: quantitative trait locus
SNP: single nucleotide polymorphism.
