# Supplementary material for: A genome-wide survey with different rapeseed ecotypes uncovers footprints of domestication and breeding
Source: J Exp Bot. 2017 Sep 11;68(17):4791–801. doi: 10.1093/jxb/erx311 (PMC5853444; doi:10.1093/jxb/erx311)
Supplement: Supplementary Figures [file erx311_suppl_supplementary_figuress1-s3-tabless3-s5-s6-s13.pdf]

Supporting Information

Fig. S1 Genome-wide patterns of linkage disequilibrium (LD) across chromosomes in the A (up) and C subgenome (down) of *Brassica napus* measured with 33,186 SNPs.

Fig. S2 The average distance of LD decay in the A and C subgenomes of *B. napus*.

Fig. S3 Venny plot of overlapped genes among three data sets in rapeseed and *Arabidopsis*.

Table S1# List of rapeseed accessions used in this study.

Table S2 Genotype data for 327 accessions in this study (http://pan.baidu.com/s/1kVFPflP).

Table S3 The distribution of SNPs across the 19 chromosomes of the rapeseed genome.

Table S4# Physical position of the weak and strong LDs

Table S5 Homeologous exchanges between the A and C subgenomes in regions for weak and strong LD.

Table S6 Sequence features of genome regions with weak and strong LD.

Table S7# Regions of selective sweep between the winter/spring and semi-winter groups in rapeseed.

Table S8# Genes within selective sweep regions between semi-winter and winter/spring groups in rapeseed.

Table S9# Functional categories of the genes in the regions of selective sweep between winter/spring and semi-winter groups in rapeseed.

Table S10# Regions of selective sweep between spring and winter groups in rapeseed.

Table S11# Genes within selective sweep regions between spring and winter groups in rapeseed.

Table S12# Functional categories of the genes in the region of selective sweep between spring and winter groups in rapeseed.

Table S13 QTLs were aligned to selective sweep within the segregation populations derived from hybrid between spring and winter rapeseed reported by Udall *et al*. (2006) and Quijada *et al*. (2006).

Table S14# A list of 4,304 genes from genome regions differentiating between winter and spring groups in rapeseed.

Table S15# A list of 4,247 differentially expressed genes between winter and spring groups in rapeseed as detected by Lu *et al*. (2014).

Table S16# A list of 5,500 differentially expressed genes between summer and winter ecotypes in *Arabidopsis thaliana* as detected by Des Marais *et al*. (2012).

Table S17# A list of 202 genes overlapping between the three data sets from rapeseed and *Arabidopsis*.

Table S18# The significant SNPs associated with flowering time across 4 years.

#available as a separate file.xlsx.

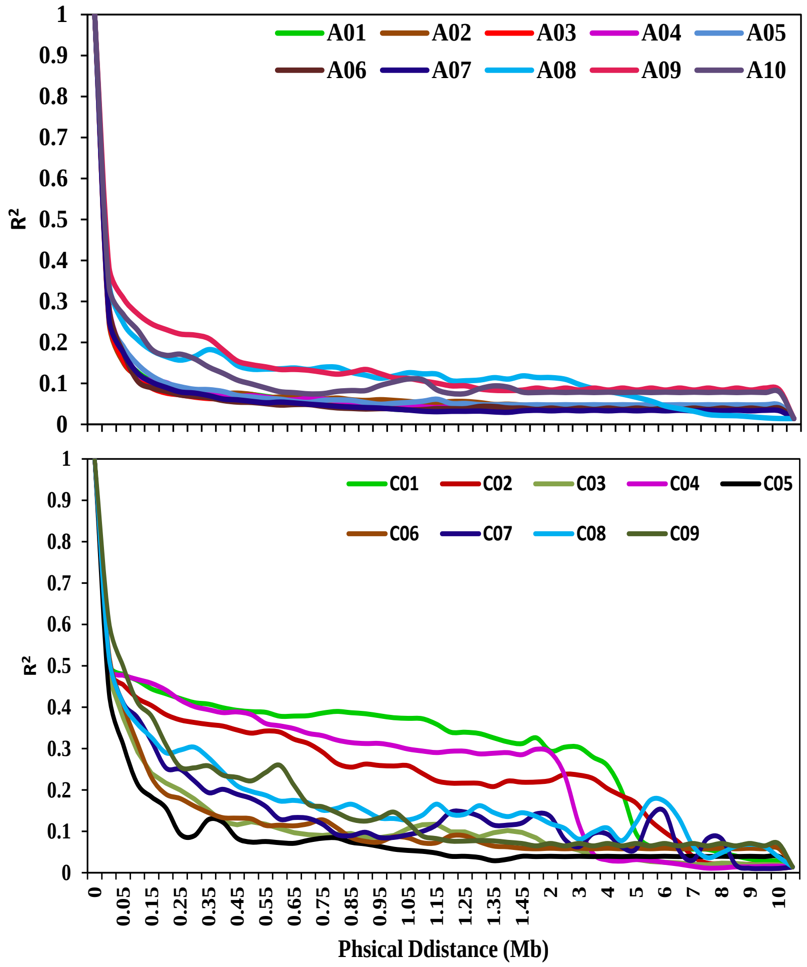

Fig. S1 Genome-wide patterns of linkage disequilibrium (LD) across chromosomes in the A (up) and C subgenome (down) of *Brassica napus*.

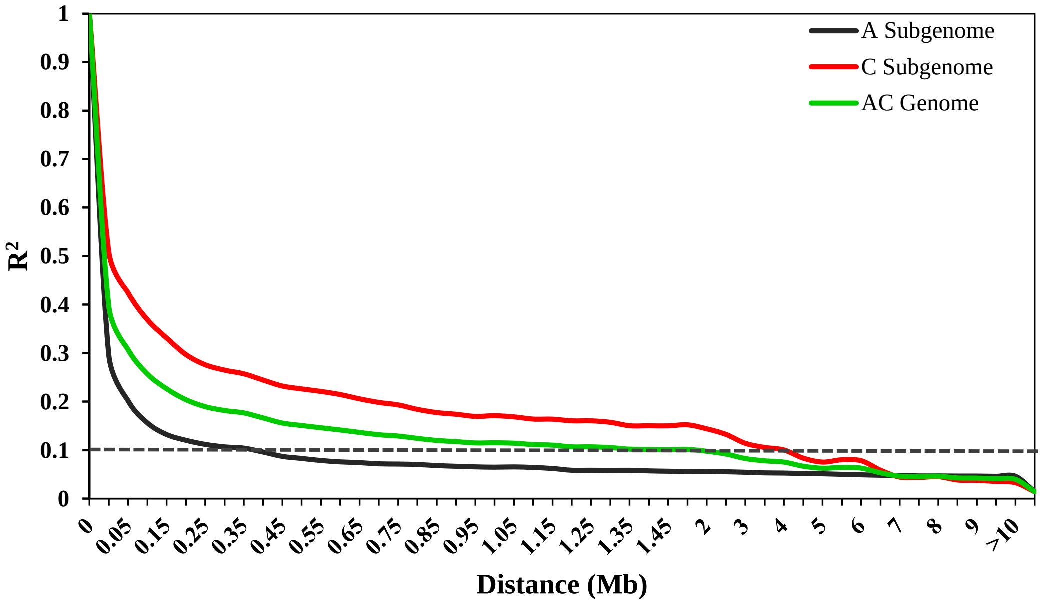

Fig. S2 The average distance of LD decay in the A and C subgenomes of *B. napus*.

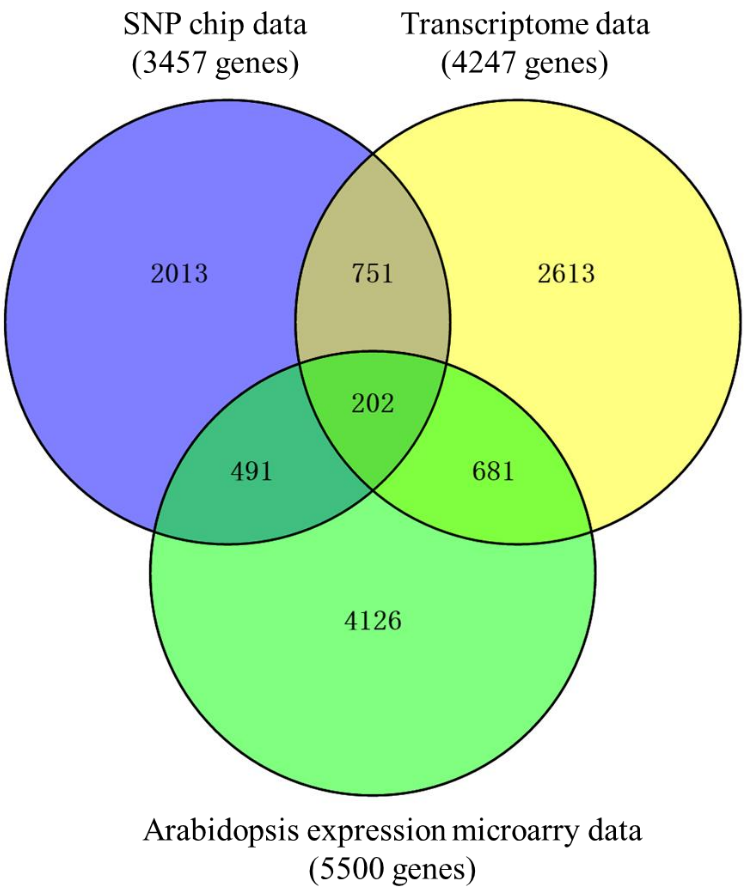

Fig. S3 Venny plot of overlapped genes among three data sets in rapeseed and *Arabidopsis*. SNP chip data including 3,457 *Arabidopsis* homologous genes (4,304 *B. napus* genes) from the top 5% Fst between winter and spring rapeseed. 4,247 DEGs (differentially expressed genes) from rapeseed detected by transcriptome sequencing and 5,500 DEGs from *Arabidopsis* detected by expression microarray analysis.

Table S3 The distribution of SNPs across the 19 chromosomes of the rapeseed genome

Chromosome	SNPs	Size (Mb)	SNPs/ 100kb	Genes/ 100kb	Average r^2 value	LD decay (Mb) (r^2 = 0.1)
A01	1700	23.24	7	15	0.082	0.15 - 0.20
A02	1493	24.79	6	14	0.100	0.10 - 0.15
A03	2416	29.73	8	18	0.072	0.10 - 0.15
A04	1689	19.14	9	14	0.086	0.20 - 0.25
A05	1838	23.02	8	15	0.096	0.20 - 0.25
A06	1783	24.39	7	15	0.079	0.10 - 0.15
A07	2160	24.01	9	15	0.079	0.15 - 0.20
A08	1373	18.93	7	15	0.150	1.15 - 1.20
A09	1745	33.86	5	15	0.151	1.15 - 1.20
A10	1796	17.38	10	16	0.122	0.45 - 0.50
C01	2511	38.77	6	10	0.396	4.50 - 5.00
C02	2353	46.19	5	10	0.280	5.50 - 6.00
C03	2828	60.56	5	12	0.138	0.60 - 0.65
C04	3254	48.89	7	11	0.313	3.00 - 3.50
C05	1116	43.09	3	11	0.110	0.20 - 0.25
C06	1466	37.19	4	11	0.138	0.80 - 0.85
C07	1882	44.77	4	11	0.144	2.00 - 2.50
C08	1697	38.39	4	12	0.173	4.00 - 4.50
C09	1141	48.5	2	10	0.162	1.05 - 1.10
A subgenome	17993	238.49	8	15	0.102	0.35 - 0.40
C subgenome	18248	406.35	4	11	0.206	3.50 - 4.00
Whole genome	36241	644.84	6	13	0.154	1.35 - 1.45

Table S5 Homeologous exchanges between the A and C subgenomes in regions for weak and strong LD

Subgenomes	No. of blocks	No. of homeologous blocks	Percentage of homeologous blocks
Weak LD regions			
A	705	267	37.90%
C	197	101	51.30%
Strong LD regions			
A	168	78	46.40%
C	445	205	46.10%

Table S6 Sequence features of genome regions with weak and strong LD

Features	weak LD regions			strong LD regions		
	A	C	AC	A	C	AC
No. of blocks	705	197	902	168	445	613
Block size(Mb)	3.15	0.76	3.91	0.43	1.63	2.06
Retroelements (%)	3.50	5.51	3.88	11.69	18.05	16.67
DNA Transposons (%)	1.06	2.27	1.29	2.02	2.79	2.63
GC content (%)	36.77	36.09	36.64	32.84	34.01	33.77
Gene density (No. of genes/Mb)	337	292	328	197	138	150

Table S13 QTLs were aligned to selective sweep within the segregation populations derived from hybrid between spring and winter rapeseed reported by Udall *et al*. (2006) and Quijada *et al*. (2006)

QTL names	Trait	Linkage map	Start	Stop	Selective sweep regions	Reference
hsy1.4	Seed yield	A01	1,312,320	2,979,230	-	Udall et al.2006
htw1.4	Test weight	A01	2,978,823	5,827,741	W-01	Quijada et al.2006
hsw1.3	Seed weight	A01	5,827,413	16,321,255	-	Udall et al.2006
hph1.4	Plant height	A01	16,449,466	20,699,444	-	Quijada et al.2006
hdtf2.1, hph2.1, hld2.3	Days to flowering, plant height, lodging	A02	32,458	3,454,175	W-02	Udall et al.2006
hph3.1	Plant height	A03	3,508,201	6,387,727	-	Quijada et al.2006
hph3.3, hdtf3.2	Plant height, days to flowering	A03	4,992,796	9,624,656	-	Quijada et al.2006
hdtf3.3	Days to flowering	A03	4,992,796	7,979,595	-	Udall et al.2006
hph3.5, htw3.3	Plant height, test weight	A03	15,547,362	20,933,851	S-08	Udall et al.2006
hsy3.3	Seed yield	A03	20,933,597	27,149,934	-	Udall et al.2006
hsw5.5	Seed weight	A05	19,236,244	21,080,264	W-07	Udall et al.2006
hph6.5	Plant height	A06	582,317	2,916,544	-	Udall et al.2006
hdtf6.4	Days to flowering	A06	16,012,424	19,675,822	-	Udall et al.2006
hph7.3	Plant height	A07	861,231	6,968,437	-	Quijada et al.2006
hdtf7.5, hph7.3; hsy7.4	Days to flowering, plant height, seed yield	A07	11,205,929	13,444,905	-	Udall et al.2006; Quijada et al.2006
hsy7.2	Seed yield	A07	13,444,128	14,751,299	-	Quijada et al.2006
hdtf9.1	Days to flowering	A09	2,809,819	8,636,824	-	Quijada et al.2006
hsy10.3	Seed yield	A10	1,359,823	6,116,215	W-19, S-10	Udall et al.2006
hsy10.2, hld10.4	Seed yield, lodging	A10	6,115,478	9,869,889	-	Udall et al.2006
hph10b.3, hsy10.2; hsy10.4	Plant height, seed yield	A10	9,869,157	13,259,907	-	Quijada et al.2006; Udall et al.2006

hph11.3	Plant height	C01	3,065,649	3,319,900	-	Udall et al.2006
hdtf12a.1, hph12a.3, hdtf12a.3; hdtf12.3, hph12.3, hdtf12.5,	Days to flowering, plant height	C02	210,364	6,707,092	-	Quijada et al.2006; Udall et al.2006
hdtf12.4	Days to flowering	C02	5,566,513	6,707,092	-	Udall et al.2006
hdtf12b.1, hph12b.1, hsy12.1	Days to flowering, plant height, seed yield	C02	18,682,450	31,673,880	from W-22 to W-30	Quijada et al.2006
hdtf12.4	Days to flowering	C02	42,040,528	42,121,017	-	Udall et al.2006
hsy13.3	Seed yield	C03	1,814,372	5,734,971	-	Quijada et al.2006
hdtf13a.3	Days to flowering	C03	9,154,164	12,284,613	-	Udall et al.2006
hsw13.3, hsy13.3	Seed weight, seed yield	C03	17,837,356	23,464,136	W-33	Udall et al.2006
hdtf13.4	Days to flowering	C03	21,268,069	29,017,005	W-33, S-16, S-17	Udall et al.2006
htw13.1	Test weight	C03	23,463,567	29,017,005	S-16, S-17	Quijada et al.2006
hdtf13b.3	Days to flowering	C03	32,464,543	35,072,240	-	Udall et al.2006
hsy15.2	Seed yield	C05	7,497,270	8,114,484	-	Quijada et al.2006
hoil15.1	Oil content	C05	24,720,692	32,521,022	-	Udall et al.2006
htw15.2, htw15.4	Test weight	C05	39,500,896	41,027,393	W-38, W-39	Quijada et al.2006
htw16.4	Test weight	C06	9,571,031	16,634,369	W-40, S-20, S-21	Udall et al.2006
hoil17.1	Oil content	C07	16,194,996	27,842,805	W-44, W-45, W-46, S-22, S-23	Udall et al.2006
hld19.3	lodging	C09	8,497,788	17,390,896	W-50, W-51, S-26, S-27	Udall et al.2006
hsw19.3	Seed weight	C09	37,799,232	43,593,715	W-53	Quijada et al.2006
hph19.5, hdtf19.5	Plant height, days to flowering	C09	43,171,116	46,370,507	-	Udall et al.2006
